# 10-Year Trends in Serum Lipid Levels and Dyslipidemia Among Children and Adolescents From Several Schools in Beijing, China

**DOI:** 10.2188/jea.JE20140252

**Published:** 2016-12-05

**Authors:** Wenqing Ding, Hong Cheng, Yinkun Yan, Xiaoyuan Zhao, Fangfang Chen, Guimin Huang, Dongqing Hou, Jie Mi

**Affiliations:** 1 Department of Epidemiology, Capital Institute of Pediatrics, Beijing, China; 2 Department of Maternal and Child Health Care, School of Public Health, Ningxia Medical University, Yinchuan, China

**Keywords:** serum lipids, trend, dyslipidemia, obesity, prevalence

## Abstract

**Background:**

Serum lipid trends in children and adolescents are predictors of the future prevalence of cardiovascular disease in adults.

**Methods:**

Data were obtained from cross-sectional studies conducted in 2004 and 2014. A total of 3249 children aged 6–18 years were included in the present study; serum total cholesterol (TC), high-density lipoprotein cholesterol (HDL-C), low-density lipoprotein cholesterol (LDL-C), and triglycerides (TG) were measured.

**Results:**

Overall, upward trends in mean TC, non-HDL-C, and LDL-C levels, and in geometric mean TG levels, were observed (all *P* < 0.001). Mean HDL-C levels significantly decreased between 2004 and 2014 (from 1.54 mmol/L to 1.47 mmol/L; *P* < 0.001). The prevalence of abnormal levels of serum lipids, with the exception of the prevalence of low HDL-C (*P* = 0.503), significantly increased over the study period (all *P* < 0.05). The prevalence of hyperlipidemia (from 13.3%; 95% confidence interval [CI], 11.6%–15.0% to 24.5%; 95% CI, 22.4%–26.6%; *P* < 0.001) and dyslipidemia (from 18.8%; 95% CI, 16.9%–20.7% to 28.9%; 95% CI, 26.7%–31.3%; *P* < 0.001) also increased from 2004 to 2014. The prevalence of abnormal serum lipids increased, and mean serum lipid levels, with the exception of TC levels, worsened in subjects with obesity compared with non-overweight subjects, as well as in subjects with mixed obesity compared with non-obese subjects (*P* < 0.05 for all).

**Conclusions:**

Adverse trends in serum lipid concentrations over the past 10 years were observed among children aged 6–9 years, with the exception of specific lipids, and among adolescents aged 10–18 years, from several schools in Beijing, China.

## INTRODUCTION

In China, cardiovascular disease (CVD) has become a leading cause of death over the past two decades as a result of economic growth and associated changes in lifestyle and diet, including unhealthy dietary patterns and an increase in the prevalence of overweight.^1^^,^^2^ For example, the total mortality resulting from CVD increased from 240.03 to 268.92 per 100 000 individuals between 2004 and 2010, for an average annual increase of 2.17%.^3^ Dyslipidemia is one of the strongest traditional risk factors for the development of CVD, and it often emerges during childhood and adolescence.^4^ A correlation between serum lipid and lipoprotein levels in childhood and levels in adulthood has also been demonstrated.^5^ Moreover, a cohort study demonstrated that low-density lipoprotein cholesterol (LDL-C) levels during childhood are associated with carotid artery intima-media thickness in adults.^6^ Exposure to high levels of LDL-C in childhood may contribute to the development of atherosclerosis in adulthood.^7^ Thus, recent trends in serum lipids in children and adolescents are likely to be important predictors of subsequent CVD trends in adults.

Data from a nationally representative cross-sectional study conducted from 2007 to 2008 revealed a high prevalence of high total cholesterol (TC), high LDL-C, and low high-density lipoprotein cholesterol (HDL-C) in the adult Chinese population. However, awareness levels, treatment rates, and control rates for hypercholesterolemia were low. Without effective intervention, prevalence of atherosclerotic CVD may soar in the near future in China.^8^ In addition, dyslipidemia is positively associated with obesity and predicts the majority of the increased CVD risk observed in obese subjects.^9^ Moreover, obesity in adults, children, and adolescents has been rising rapidly over the past two decades in China.^10^^,^^11^ Thus, it is important to obtain data on trends in serum lipid levels among children and adolescents over the past decade and, based on these data, to take early measures to control dyslipidemia throughout youth and into adulthood.

Over the past decade, Beijing, one of the most urbanized cities in China, has also been experiencing rapid increases in the prevalence of CVD and other chronic diseases and in the burdens resulting from these diseases. However, to our knowledge, no previous study has examined secular trends in blood lipid parameters among children and adolescents. Thus, the objectives of the present study were: (1) to provide the most up-to-date data on serum lipid levels in the general population of children and adolescents in Beijing; (2) to estimate trends in serum lipid levels among Chinese children and adolescents from 2004 to 2014; and (3) to examine the relationship between serum lipid concentrations and obesity.

## METHODS

### Study population

Data were obtained from two cross-sectional studies. The 2004 data were obtained from the Beijing Child and Adolescent Metabolic Syndrome (BCAMS) study; the design of this study and the data collected for it have been described previously.^12^^,^^13^ The BCAMS study comprised a questionnaire, anthropometric measurements, and a finger capillary blood test administered to a large, representative sample of 19 593 children aged 6–18 years (50% boys) from schools in Beijing in 2004. To understand blood lipid levels among Beijing’s school-age children, a subsample of 1660 children aged 6–18 years was selected by convenience sampling from the four classes in each grade in one 3-year, full-time public high school, one elementary school, and one middle school. Furthermore, venipuncture blood samples were collected from these samples, and serum levels of triglycerides (TG), TC, HDL-C, and LDL-C were measured. For the 2014 study, 1649 subjects aged 6–18 years were selected from the same regions from which the 2004 study subjects were drawn, and the 2014 study employed methods similar to those used in 2004.

Subjects who had blood samples drawn, for whom complete information from a physical assessment were available, and who had lived in Beijing with their parents or guardians for at least 6 months were eligible for inclusion in the study. Children and adolescents (aged 6–18 years) with cancer, endocrine diseases, metabolic diseases, or serious liver or kidney disease were excluded from the study.

Signed informed consent was obtained from all participants and/or their parents or guardians throughout the study. The Ethics Committee of the Capital Institute of Pediatrics in Beijing approved the study protocol.

### Anthropometric measurements

Anthropometric data were collected by trained research staff according to a standardized protocol. Standing heights without shoes were measured to the nearest 0.1 cm using wall-mounted stadiometers. Weights were measured to the nearest 0.1 kg using beam scales that were accurate to a maximum weight of 140 kg; the children wore light, indoor clothing when they were measured. Waist circumferences were measured to the nearest 0.1 cm at the end of normal expiration in a horizontal plane at a point midway between the last rib and the crest of the ilium using non-elastic tape. Both surveys employed similar data collection methods. Body mass index (BMI) was calculated as the weight in kilograms divided by the square of the height in meters.

### Laboratory tests

Blood samples were collected from all participants after an overnight fast (>12 hours). A plasma sample was stored at −80°C until further analysis. The measurements of serum lipids in the two surveys were both performed in the Clinical Laboratory of the Capital Institute of Pediatrics in Beijing, China. Serum TG and TC levels were measured using enzymatic methods (Roche, Basel, Switzerland in 2004; Olympus, Tokyo, Japan in 2014). HDL-C and LDL-C levels were measured directly using a Hitachi 7060 chemistry analyzer (Hitachi, Tokyo, Japan) in 2004 and an Olympus AU640 automatic chemistry analyzer (Olympus, Tokyo, Japan) in 2014. Although different chemistry analyzers and methods were used during the two surveys in which serum samples were tested, serum lipid measurements were standardized based on the criteria of a lipid measurement standardization program. In addition, the laboratory that performed the measurements participated in the External Quality Assessment (EQA) program of the National Center for Clinical Laboratories in China. Intra-laboratory quality control was performed, and the measurements were within the acceptable limits of bias and imprecision specified by the lipid measurement standardization program. For example, both the bias and imprecision of serum TG levels were not greater than 5%, and the overall error was less than 15%.^14^ The laboratory procedures followed in 2014 were identical to those used in 2004. Therefore, the standardization program ensured that serum lipids measurements were accurate and comparable across surveys.^15^

### Definitions of abnormal serum lipid levels

Abnormal lipid profiles were defined according to the 2011 Expert Panel on Integrated Guidelines for Cardiovascular Health and Risk Reduction in Children and Adolescents: TG ≥1.13 mmol/L for children aged 6–9 years, TG ≥1.47 mmol/L for children aged 10–19 years, TC ≥5.18 mmol/L, LDL-C ≥3.37 mmol/L and HDL-C ≤1.04 mmol/L. Because non-HDL-C levels have been shown to be more predictive of persistent dyslipidemia (and, therefore, of atherosclerosis and future events) than TC, LDL-C, or HDL-C levels alone, the prevalence of high non-HDL-C levels is also presented. The non-HDL-C level was calculated as the TC level minus the HDL-C level. High non-HDL-C levels were defined as non-HDL-C ≥3.76 mmol/L.^16^ Hyperlipidemia was defined by the presence of high TG or high TC levels. Dyslipidemia was defined as the presence of any one of the following four factors: high LDC-C levels, low HDL-C levels, high TG levels, or high TC levels.

### Obesity status

Overweight and obesity were defined based on age- and sex-specific BMI cutoffs recommended by the Working Group on Obesity in China.^17^ Abdominal obesity was defined as a waist circumference equal to or greater than that at the 90th percentile for Chinese children and adolescents of the same age and sex.^18^ Obesity types were defined according to a combination of BMI and waist circumference. Peripheral obesity was defined as the presence of a high BMI but a normal waist circumference, abdominal obesity was defined as the presence of a high waist circumference but a normal BMI, and mixed obesity was defined as the presence of both a high BMI and a high waist circumference.^19^

### Statistical analysis

Means and standard errors are presented for the continuous variables, and percentages and numbers are given for the categorical variables. Serum triglyceride concentrations are presented as geometric means and standard errors because the distribution of TG is skewed. The prevalence of abnormal serum lipid levels is also shown. Comparisons were performed using a chi-squared test for the categorical variables and a two-sample *t*-test for the continuous variables. Because the age distribution of the subjects differed substantially between the two study periods, the differences in BMI and waist circumference between the two periods were analyzed using a general linear model adjusted for age, and comparisons of the extents and types of obesity over time were analyzed after stratifying by age. Whether time trends were significant was determined using multivariate logistic regression analysis for prevalence data and using linear regression analysis for continuous variables, with adjustments for age and sex. A value of *P* < 0.05 was considered statistically significant. All statistical analyses were performed using SPSS software version 13.0 (SPSS, Inc., Chicago, IL, USA).

## RESULTS

Table 1 shows the characteristics of the study populations in 2004 and 2014. The sex distribution remained stable over time. As expected, the age-stratified prevalence of overweight, obesity, and the different types of obesity, increased over time.

**Table 1.** Characteristics of children and adolescents in Beijing, 2004 and 2014

	2004	2014	*P value*
*n*	1600	1649	
Mean (SE) age, years	11.9 (0.1)	11.4 (0.1)	<0.001
Boys	884 (55.3)	895 (54.3)	0.577
Mean (SE) BMI, kg/m^2^	18.9 (0.9)	19.4 (0.1)	<0.001^a^
Mean (SE) WC, cm	65.2 (0.3)	67.6 (0.3)	<0.001^a^
Overweight^b^			
6–9 years	10.6 (7.3–14.0)	14.6 (11.3–17.9)	0.091
10–18 years	9.7 (7.9–11.3)	22.1 (19.4–24.9)	<0.001
Obesity^b^			
6–9 years	11.2 (7.9–14.5)	19.4 (16.1–22.9)	0.002
10–18 years	7.6 (6.0–9.1)	15.7 (13.3–18.3)	<0.001
Abdominal obesity^b^			
6–9 years	15.3 (11.8–18.9)	24.7 (21.1–28.1)	<0.001
10–18 years	13.5 (11.6–15.4)	31.4 (28.4–34.4)	<0.001
Types of obesity			
Peripheral obesity	0.3 (0.1)	0.6 (0.2)	<0.001^c^
Abdominal obesity	6.5 (0.6)	15.2 (0.9)	
Mixed obesity	7.4 (0.7)	13.7 (0.9)	

BMI, body mass index; WC, waist circumference; SE, standard error of mean.

Comparisons were performed using chi-squares for sex and a two-sample *t* test for age.

^a^The difference between 2004 and 2014 was tested using a general linear model adjusted for age and sex.

^b^Reported as % (95% confidence interval).

^c^The difference between 2004 and 2014 was tested using a logistic regression model adjusted for age and sex.

Trends in mean serum lipid levels, stratified by age group and sex for each survey period, are presented in Table 2. Among all study participants, the mean serum TC level significantly increased from 4.04 mmol/L to 4.25 mmol/L, the geometric mean serum TG level increased from 0.81 mmol/L to 0.88 mmol/L, non-HDL-C levels increased from 2.50 mmol/L to 2.77 mmol/L, and LDL-C level increased from 2.23 mmol/L to 2.35 mmol/L between 2004 and 2014 (*P* < 0.001 for all). After being stratified by sex and age, trends in the mean serum TC, TG, LDL-C, and non-HDL-C levels were similar to the overall trends for the entire study population and consistent with an upward trend, although each trend was not always significant in each group and the magnitudes of the trends varied. Overall, the mean serum HDL-C level decreased significantly from 1.54 mmol/L to 1.47 mmol/L (*P* < 0.001). A significant decreasing trend was observed for HDL-C in children aged 10–18 years (*P* < 0.01) but not in children aged 6–9 years (*P* = 0.331 for boys and *P* = 0.684 for girls).

**Table 2.** Mean serum lipid concentrations over time by sex and age among children and adolescents in Beijing, 2004–2014

	TC			TG^a^			HDL-C			Non-HDL-C			LDL-C		
				
	2004	2014	*P value*	2004	2014	*P value*	2004	2014	*P value*	2004	2014	*P value*	2004	2014	*P value*
**All**	4.04 (0.02)	4.25 (0.02)	<0.001	0.81 (0.01)	0.88 (0.01)	<0.001	1.54 (0.01)	1.47 (0.02)	<0.001	2.50 (0.02)	2.77 (0.02)	<0.001	2.23 (0.01)	2.35 (0.02)	<0.001
**Boys**	4.00 (0.02)	4.21 (0.02)	<0.001	0.78 (0.01)	0.87 (0.01)	<0.001	1.53 (0.01)	1.46 (0.01)	<0.001	2.47 (0.02)	2.76 (0.02)	<0.001	2.23 (0.02)	2.34 (0.02)	<0.001
Age, years															
6–9	4.20 (0.05)	4.43 (0.04)	<0.001	0.76 (0.03)	0.80 (0.02)	0.907	1.63 (0.02)	1.59 (0.02)	0.331	2.55 (0.04)	2.84 (0.03)	<0.001	2.36 (0.04)	2.41 (0.03)	0.329
10–18	3.94 (0.03)	4.11 (0.03)	<0.001	0.79 (0.02)	0.91 (0.02)	<0.001	1.49 (0.01)	1.38 (0.01)	<0.001	2.45 (0.03)	2.72 (0.03)	<0.001	2.18 (0.02)	2.31 (0.03)	<0.001
**Girls**	4.08 (0.02)	4.29 (0.03)	<0.001	0.85 (0.01)	0.89 (0.01)	0.038	1.55 (0.01)	1.49 (0.01)	<0.001	2.52 (0.02)	2.79 (0.02)	<0.001	2.25 (0.02)	2.36 (0.02)	<0.001
Age, years															
6–9	4.11 (0.05)	4.39 (0.04)	<0.001	0.81 (0.03)	0.83 (0.03)	0.266	1.56 (0.03)	1.53 (0.02)	0.684	2.55 (0.04)	2.87 (0.04)	<0.001	2.33 (0.04)	2.44 (0.04)	0.080
10–18	4.06 (0.03)	4.23 (0.03)	<0.001	0.87 (0.02)	0.92 (0.02)	0.006	1.55 (0.01)	1.47 (0.01)	<0.001	2.52 (0.02)	2.75 (0.03)	<0.001	2.23 (0.02)	2.32 (0.03)	0.004

HDL-C, high-density lipoprotein cholesterol; TC, total cholesterol; LDL-C, low-density lipoprotein cholesterol; TG, triglycerides. Non-HDL-C levels equal serum TC levels minus HDL-C. Data are presented as mean (SE). Linear trends in mean serum lipid concentrations were tested using a multivariate linear regression model adjusted for sex and age.

^a^The distribution of TG is skewed. Data are presented as geometric means (SE).

Table 3 shows the prevalence of abnormal serum lipids levels over time by sex and age among children and adolescents. Overall, the prevalence of abnormal serum lipid levels increased significantly from 2004 to 2014, with the exception of the prevalence of low HDL-C levels (*P* = 0.503). For example, the prevalence of high TG increased from 9.1% (95% CI, 7.7%–10.5%) in 2004 to 16.1% (95% CI, 14.3%–17.9%) in 2014 (*P* < 0.001). Similar trends were observed in all subgroups defined by age and sex, although this observation did not reach statistical significance in some subgroups. The prevalence of high TG significantly increased from 2004 to 2014 among children aged 10–18 years (*P* < 0.01) but did not increase among children aged 6–9 years (*P* = 0.134 for boys and *P* = 0.117 for girls).

**Table 3.** Prevalence of abnormal serum lipids over time by sex and age among children and adolescents in Beijing, 2004–2014

	High TC			High TG			Low HDL-C			High non-HDL-C		
			
	2004	2014	*P value*	2004	2014	*P value*	2004	2014	*P value*	2004	2014	*P value*
**All**	4.9 (3.8–6.0)	10.7 (9.2–12.2)	<0.001	9.1 (7.7–10.5)	16.1 (14.3–17.9)	<0.001	6.0 (4.8–7.2)	6.4 (5.2–7.6)	0.503	2.8 (2.0–3.6)	7.6 (6.3–8.9)	<0.001
**Boys**	5.2 (3.7–6.7)	10.7 (8.7–12.7)	<0.001	8.5 (6.6–10.4)	15.4 (13.0–17.8)	<0.001	6.8 (5.1–8.5)	8.5 (6.7–10.3)	0.152	3.2 (2.0–4.4)	7.0 (5.3–8.7)	0.001
Age, years												
6–9	6.3 (3.4–9.7)	14.8 (10.4–18.9)	0.006	13.5 (9.2–18.4)	17.0 (12.9–21.5)	0.134	1.4 (0.0–3.4)	2.5 (0.9–4.4)	0.408	2.9 (1.0–5.3)	7.3 (4.4–10.4)	0.054
10–18	4.7 (3.2–6.5)	8.5 (6.2–10.9)	0.003	6.9 (5.1–8.9)	14.4 (11.8–27.3)	<0.001	8.5 (6.3–10.6)	11.8 (9.2–14.6)	0.257	3.3 (2.1–4.8)	6.9 (5.0–9.0)	0.003
**Girls**	4.7 (3.1–6.3)	10.7 (8.5–12.9)	<0.001	10.2 (8.0–12.4)	17.0 (14.3–19.7)	0.001	5.0 (3.4–6.6)	3.8 (2.4–5.2)	0.317	2.3 (1.2–3.4)	8.4 (6.4–10.4)	<0.001
Age, years												
6–9	3.9 (1.3–7.1)	13.5 (9.8–17.5)	0.005	16.9 (11.7–23.4)	22.5 (17.5–27.3)	0.117	5.8 (2.6–7.9)	2.9 (1.1–5.1)	0.129	1.3 (0.0–3.2)	9.1 (5.8–12.7)	0.008
10–18	4.9 (3.1–6.9)	9.2 (6.7–11.9)	0.006	8.3 (6.2–10.7)	13.8 (10.9–16.9)	0.003	4.7 (3.1–6.5)	4.4 (2.5–6.1)	0.801	2.5 (1.3–4.0)	7.9 (5.4–10.4)	0.001



	High LDL-C			Hyperlipidemia^a^			Dyslipidemia^b^					
		
	2004	2014	*P value*	2004	2014	*P value*	2004	2014	*P value*			

**All**	3.4 (2.5–4.3)	5.8 (4.7–6.9)	0.002	13.3 (11.6–15.0)	24.5 (22.4–26.6)	<0.001	18.8 (16.9–20.7)	28.9 (26.7–31.3)	<0.001			
**Boys**	3.9 (2.6–5.2)	5.3 (3.8–6.8)	0.238	12.8 (10.6–15.0)	24.4 (21.6–27.2)	<0.001	18.6 (16.0–21.2)	30.0 (27.0–33.0)	<0.001			
Age, years												
6–9	3.9 (1.4–6.8)	5.7 (3.2–8.5)	0.470	18.4 (13.0–24.1)	30.6 (25.6–35.6)	0.001	19.3 (14.0–24.5)	32.2 (27.1–37.5)	0.001			
10–18	3.8 (2.3–5.3)	5.0 (3.3–6.9)	0.202	10.9 (8.6–13.4)	20.8 (17.7–24.1)	<0.001	18.3 (15.1–21.1)	29.1 (25.3–32.9)	<0.001			
**Girls**	2.8 (1.6–4.0)	6.4 (4.7–8.1)	0.002	14.0 (11.4–16.6)	24.7 (21.6–27.8)	<0.001	17.3 (14.5–20.1)	27.3 (24.1–30.5)	<0.001			
Age, years												
6–9	2.6 (0.6–5.2)	6.9 (4.0–9.8)	0.055	20.1 (14.3–27.3)	32.0 (26.2–37.1)	0.006	21.4 (15.6–28.6)	33.8 (28.0–39.3)	0.004			
10–18	2.9 (1.6–4.3)	6.1 (4.0–8.1)	0.023	12.3 (9.6–15.2)	20.5 (16.9–24.0)	<0.001	16.1 (13.2–19.3)	23.6 (19.8–27.3)	0.002			

HDL-C, high-density lipoprotein cholesterol; TC, total cholesterol; LDL-C, low-density lipoprotein cholesterol; TG, triglycerides. Non-HDL-C levels equal serum TC minus HDL-C levels. Data are presented as prevalence (95% CI). Linear trends in the prevalence of abnormal serum lipids were tested using a multivariate logistic regression model adjusted for sex and age.

^a^Hyperlipidemia: the presence of high TG or high TC levels.

^b^Dyslipidemia: the presence of any one of the following four factors: high LDC-C, Low HDL-C, high TG or high TC levels.

Among the four serum lipid markers studied, abnormal levels of TG were the most prevalent; the prevalence of high TG levels increased by 7.1% from 2004 to 2014. Upward trends in the prevalence of both hyperlipidemia and dyslipidemia were observed from 2004 to 2014 (*P* < 0.001 for both increases), with the prevalence of hyperlipidemia rising from 13.3% (95% CI, 11.6%–15.0%) to 24.5% (95% CI, 22.4%–26.6%) and that of dyslipidemia rising from 18.8% (95% CI, 16.9%–20.7%) to 28.9% (95% CI, 26.7%–31.3%) (*P* < 0.001 for both).

The associations between serum lipid concentrations and obesity based on the 2014 data are presented in Table 4. All of the mean serum lipid levels except for TC were significantly higher in obese or overweight subjects than in non-overweight subjects, while HDL-C levels were higher in non-overweight subjects (*P* < 0.05 for all). Obese subjects had higher serum lipid concentrations, except for mean serum TC levels, than did overweight subjects (*P* < 0.05 for all). Compared with subjects who were non-obese, abdominally obese subjects had higher mean serum TG, LDL-C, and non-HDL-C, as well as lower HDL-C levels (*P* < 0.05 for all). The mean serum TG and LDL-C levels increased, while the mean serum HDL-C levels decreased in children with peripheral obesity, abdominal obesity, and mixed obesity (*P* < 0.001 for all). Subjects with mixed obesity had higher mean serum TG, LDL-C, and non-HDL-C levels, and lower HDL-C levels, than did subjects with abdominal obesity and peripheral obesity, respectively obesity (*P* < 0.05 for all). Subjects with peripheral obesity had lower mean serum TG, LDL-C, and higher HDL-C levels than did subjects with abdominal. (*P* < 0.05 for all).

**Table 4.** Mean serum lipid concentrations among children and adolescents obesity extent and type

	TC	TG^a^	HDL-C	LDL-C	Non-HDL-C
BMI					
Non-overweight	4.25 (0.02)	0.82 (0.01)	1.53 (0.01)	2.30 (0.02)	2.72 (0.02)
Overweight	4.21 (0.05)	0.93 (0.04)*	1.38 (0.02)*	2.40 (0.04)*	2.82 (0.04)*
Obesity	4.33 (0.05)	1.16 (0.04)*^¥^	1.33 (0.02)*^¥^	2.55 (0.04)*^¥^	3.01 (0.05)*^¥^
***P value for trend***	**0.329**	**<0.001**	**<0.001**	**<0.001**	**<0.001**
WC					
Non-obese	4.25 (0.02)	0.81 (0.01)	1.53 (0.01)	2.30 (0.02)	2.72 (0.02)
Abdominal obesity	4.26 (0.03)	1.07 (0.03)	1.34 (0.01)	2.48 (0.03)	2.92 (0.03)
***P value***	**0.365**	**<0.001**	**<0.001**	**<0.001**	**<0.001**
Types of obesity					
Non-obese	4.24 (0.02)	0.81 (0.01)	1.53 (0.01)	2.30 (0.02)	2.72 (0.02)
Peripheral obesity	4.45 (0.18)	0.97 (0.18)	1.52 (0.11)	2.39 (0.15)	2.93 (0.18)
Abdominal obesity	4.21 (0.05)	0.98 (0.04)^Δ§^	1.36 (0.02)^Δ§^	2.42 (0.04)^Δ§^	2.84 (0.04)^Δ^
Mixed obesity	4.33 (0.05)	1.17 (0.04)^Δ#§^	1.32 (0.02)^Δ#§^	2.56 (0.04)^Δ#§^	3.01 (0.05)^Δ#§^
***P value for trend***	**0.520**	<0.001	<0.001	**<0.001**	**<0.001**

BMI, body mass index; WC, waist circumference; HDL-C, high-density lipoprotein cholesterol; TC, total cholesterol; LDL-C, low-density lipoprotein cholesterol; TG, triglycerides. Non-HDL-C levels equal serum TC levels minus HDL-C levels. Data are presented as means (SEs).

^a^The TG distribution is skewed. Data are presented as geometric means (SEs). Differences between mean serum lipid concentrations by obesity extents and types were tested using a general linear model with that adjusted for sex and age.

*P < 0.05 compared with non-overweight group.

^¥^*P* < 0.05 compared with overweight group.

^Δ^*P* < 0.05 compared with non-obese group.

^#^*P* < 0.05 compared with abdominal obesity group.

^§^*P* < 0.05 compared with peripheral obesity group.

The prevalence of abnormal levels of serum lipids, with the exception of high TC levels, was higher in obese children than in non-overweight subjects (*P* < 0.01 for all). The prevalence of high TG, low HDL-C, and high non-HDL-C levels was greater in obese children than in overweight groups (*P* < 0.05 for all). The prevalence of both high TG and low HDL-C levels was higher in the overweight groups than in the non-overweight groups (*P* < 0.05 for both). All of the abnormal serum lipid levels, except for high TC levels, were significantly more prevalent in abdominally obese subjects than in non-obese subjects (*P* < 0.05 for all).

The prevalence of high TG, high LDL-C, and high non-HDL-C levels increased in children with peripheral obesity; such prevalence increased more in children with mixed obesity than in other children and decreased in children with abdominal obesity (*P* < 0.001). The prevalence of low HDL-C levels increased in children with peripheral, abdominal, and mixed obesity (*P* < 0.001). The prevalence of abnormal levels of serum lipids, with the exception of high TC levels, was higher in the groups with abdominal obesity and mixed obesity compared with the non-obese group (*P* < 0.05 for all). The prevalence of high TG and low HDL-C levels was higher in the group with mixed obesity than in the abdominal obesity group (*P* < 0.05 for both; Table 5).

**Table 5.** Prevalence of abnormal levels of serum lipids among children and adolescents by obesity extent and type

	High TC	High TG	Low HDL-C	High LDL-C	High non-HDL-C
BMI					
Non-overweight	10.9 (9.1–12.7)	11.4 (9.6–13.5)	3.1 (2.1–4.1)	4.8 (3.6–6.2)	6.1 (4.7–7.7)
Overweight	10.0 (6.3–13.7)	18.5 (14.0–23.5)*	12.3 (8.6–16.4)*	6.2 (3.5–9.1)	8.1 (4.9–11.2)
Obesity	12.1 (8.3–16.7)	37.2 (30.9–43.3)*^¥^	13.9 (9.3–18.3)*^¥^	9.9 (6.2–13.8)*	14.8 (10.5–19.8)*^¥^
***P value for trend***	**0.770**	**<0.001**	**<0.001**	**0.013**	**<0.001**
WC					
Non-obese	10.3 (8.6–12.0)	10.9 (9.0–12.8)	3.4 (2.4–4.5)	4.4 (3.2–5.7)	5.8 (4.5–7.3)
Abdominal obesity	12.2 (9.3–15.4)	29.6 (25.5–33.8)	13.1 (9.8–16.1)	9.1 (6.4–12.0)	11.9 (9.0–15.1)
***P value***	**0.210**	**<0.001**	**<0.001**	**<0.001**	**<0.001**
Types of obesity					
Non-obese	10.3 (8.4–12.3)	10.7 (8.9–12.7)	3.3 (2.3–4.5)	4.3 (3.2–5.6)	5.7 (4.3–7.0)
Peripheral obesity	11.1 (0.0–37.5)	33.3 (0.0–66.7)^Δ^	11.1 (0.0–33.3)^Δ^	11.1 (0.0–37.5)	22.2 (0.0–50.0)
Abdominal obesity	12.2 (8.2–16.7)	22.7 (17.7–28.2)^Δ^	12.2 (8.1–16.9)^Δ^	8.4 (5.0–12.2)^Δ^	9.7 (6.1–13.4)^Δ^
Mixed obesity	12.1 (7.9–16.7)	37.4 (30.7–43.9)^Δ#^	14.0 (9.7–18.9)^Δ#^	9.8 (6.3–14.1)^Δ^	14.5 (10.0–19.4)^Δ^
***P value for trend***	**0.775**	**<0.001**	**<0.001**	**0.003**	**<0.001**

BMI, body mass index; WC, waist circumference. HDL-C, high-density lipoprotein cholesterol; TC, total cholesterol; LDL-C, low-density lipoprotein cholesterol; TG, triglycerides. Non-HDL-C levels equal serum TC levels minus HDL-C levels. Data are presented as prevalence (95% CI). Differences between mean serum lipid concentrations by obesity extent and type of were tested using a multivariate logistic regression model adjusted for sex and age.

*P < 0.05 compared with non-overweight group.

^¥^*P* < 0.05 compared with overweight group.

^Δ^*P* < 0.05 compared with non-obese group.

^#^*P* < 0.05 compared with abdominal obesity group.

Because the prevalence of overweight, obesity, and abdominal obesity increased over time, trends in serum lipid concentration among subjects stratified by weight status defined by BMI and waist circumference were analyzed (eTable 1). Overall, among children and adolescents with either any obesity or abdominal obesity, increases in TC, non-HDL-C, and triglycerides were observed over the study period. For example, mean TC increased by 0.30 mmol/L and 0.26 mmol/L among subjects with any obesity and abdominal obesity, respectively. Among children and adolescents with any obesity or abdominal obesity, changes in HDL-C and LDL-C over time were not significant (*P* > 0.05 for both). The effects of including weight status defined by either BMI or waist circumference in the overall linear regression models while adjusting for sex and age were also estimated (eTable 2). Compared with the β coefficients estimated only with the inclusion of sex and age, those estimated also with the inclusion of weight status in the model were of a slightly smaller magnitude. For example, compared with the data for 2004, the β coefficient for TG in 2014 was 0.022 (95% CI, 0.010 to 0.033) for weight status defined by BMI.

## DISCUSSION

This article presents the first report of secular trends in serum lipid concentration in Chinese children and adolescents. Our findings revealed an adverse trend in serum lipid concentrations among children and adolescents in Beijing over the past 10 years. A previous study demonstrated that serum TC and LDL-C levels were elevated and increasing in the adult Chinese population.^8^ Mean levels of TG were found to be 128.1 mg/dL in 2000 to 2001 and 139 mg/dL in 2007 to 2008.^20^ As in adults, increasing trends in serum lipid concentrations over time were found in children and adolescents in the present study. Findings from a systematic analysis of health surveys and epidemiological studies that investigated 321 country-years of data and 3.0 million participants worldwide have demonstrated that mean TC levels declined slightly from 1980 to 2008; this decline was especially significant in high-income regions. In contrast, the mean levels of serum TC have increased in East Asian and Southeast Asian populations over this time period.^21^ Favorable trends in serum lipid concentrations were observed among youths in the United States between 1988 and 1994 and between 2007 and 2010.^22^ Moreover, investigators in Japan found no significant increasing trend in TC, non-HDL-C, or HDL-C levels in children from 1993 through 2008.^23^

Our results are inconsistent with observations made in high-income regions, such as the United States and Japan, but are consistent with previous studies of East Asia. It is possible that the increasing trend in dyslipidemia among Chinese children may follow the pattern that has been observed in developed countries, in which an initial increase in the prevalence of dyslipidemia is followed by stable or declining prevalence. This observation offers hope that we may be able to prevent further increases in the prevalence of dyslipidemia among Chinese children; however, we do not know when this increasing trend may be halted or reversed. Thus, greater focus should be placed on interventions to lower the prevalence of dyslipidemia.

It is widely recognized that differences in serum lipids levels are associated with age, sex, and race/ethnicity.^24^^,^^25^ A recent report from the National Health and Nutrition Examination Survey for 1999–2006 among United States adolescents indicated that boys tend to have higher prevalence of low HDL-C levels compared with girls, and that older youths were more likely to have abnormal lipid levels compared with younger youths.^26^ Our findings revealed adverse trends in serum lipid concentrations for girls, boys, and adolescents aged 10–18 years. However, no significant changes for specific lipids were observed among children aged 6–9 years, with the exceptions of some subgroups, for which data may have been insufficent due to low power (eTable 3). Further research, especially research performed with a larger sample, is needed to investigate the hypothesized contributions to the difference in serum lipid concentration trends between the age groups.

Previous studies have demonstrated that the ingestion of animal-based fats and obesity lead to increases in serum cholesterol levels across populations and over time.^27^^,^^28^ In recent years China has experienced a remarkable change in dietary patterns in parallel with remarkable economic progress; diets are shifting from the traditional pattern, with high intake of cereals and vegetables, to a Western pattern, with high intake of animal products and other highly energy-dense foods.^2^^,^^29^ The Chinese Health and Nutrition Surveys of 1991 through 2009 reported that the daily fat intake in children steadily increased from 54.8 g to 66.0 g and that the proportion of energy in the diet from fat increased from 21.5% to 30.0%.^30^ Although the present study did not examine dietary trends among children, the rapid transition to a high-fat diet among Chinese children between 1991 and 2009 most likely contributed to the adverse changes in serum lipid concentrations observed in the present study. In addition, lifestyle and dietary patterns have been influenced by economic growth; thus, socioeconomic factors have most likely affected the findings of the present study.^31^

Previous reports have indicated that primary cardiovascular risk factors are primarily associated with physical activity levels rather than with dietary patterns.^32^ Limited data on trends in physical activity among children and adolescents in Beijing during the study period are available. However, a recent study has reported that the daily physical activity levels of children in Beijing were insufficient and that only 15.5% of boys and 13.1% of girls participated in at least 60 minutes of “moderate-vigorous physical activities” per day.^33^ Among adults in China, physical activity levels have significantly decreased in both the occupational and domestic domains between 1991 and 2011.^34^ However, the extent to which trends in physical activity may be generalized to children and adolescents is unknown.

In China, the prevalence of overweight or obesity has been rising, especially among children and adolescents.^11^ Dyslipidemia, a frequent comorbidity of obesity, begins in childhood.^35^ The typical profile of dyslipidemia associated with obesity is a pattern of elevated TG, low HDL-C, and normal or slightly elevated LDL-C levels.^22^ In the present study, we found that all of the abnormal levels of serum lipids, with the exception of high TC, were associated with the extent and type of obesity. In addition, the prevalence of both any obesity and abdominal obesity increased over time. However, adverse trends in mean serum lipid concentrations were still observed in children and adolescents with overweight, any obesity, or abdominal obesity. Moreover, in linear regression models comparing differences in serum lipids in 2014 to 2004, the inclusion of weight status, in addition to age and sex, yielded β coefficients of a small magnitude. These findings demonstrated that serum lipids are associated with BMI, but factors other than BMI also contribute to the adverse trends in serum lipid concentrations. However, if there had been no increase in the prevalence of obesity, the adverse trends in mean serum lipid concentration over time may have been slightly smaller.

Hypertriglyceridemia and low HDL-C levels are metabolically interlinked, and this combination has been termed “atherogenic dyslipidemia”.^36^ This pattern of dyslipidemia is strongly associated with CVD in both adults and children.^37^ In our study, high TG levels, rather than high TC or high LDL-C levels, were found to have the highest prevalence among all study participants; in addition, the largest change (a 7.0% increase) was observed in the prevalence of high TG levels from 2004 to 2014. These findings suggest that preventive measures should be taken early to reduce the prevalence of abnormal serum lipids and, therefore, the burden of CVD in adults. Data from the United States National Health and Nutrition Examination Survey demonstrated that the increasing prevalence of obesity may be a factor in the observed changes in lipid levels, especially TG levels.^38^ Increased body weight and BMI have been reported to be most strongly associated with high TG and low HDL-C levels.^39^ In the present study, significant increases in the prevalence of both any obesity and abdominal obesity were also observed over the past 10 years, and obese children and adolescents were more likely to have elevated TG levels and lower HDL-C levels compared with non-obese individuals.

The main strength of this study is that it is the first to assess trends in serum lipid concentrations in Chinese children. However, the present study has several limitations. First, our data were collected in only one area of China, and our study subjects were not selected randomly from across the entire country. Although the subjects were drawn from the same school or district by convenience sampling in 2004 and 2014, the characteristics, including socioeconomic status of the student in the selected schools, could have changed over the 10 years between the surveys. Unfortunately, we could not obtain information on the characteristics of students in 2004 and 2014. Therefore, the increase of lipid levels might be due to the differences in the distribution of the students’ characteristics. Second, using BMI to define obesity does not discriminate between lean and fat body mass. Therefore, subjects of short stature or those with a muscular build may be misclassified. Moreover, using BMI may underestimate the prevalence of obesity. Waist circumference is correlated with visceral tissue, but it does not take into consideration the distribution of fat throughout the body. There is no international agreement on the cut-off points for defining obesity using an indicator of total body fat percentage. However, BMI has been used widely in many other studies because the methodology for measuring the BMI is simple, and waist circumference is considered to be a valid indicator of abdominal obesity. To facilitate comparisons between our results and the results of other studies, we analyzed the association between obesity and dyslipidemia using BMI to define obesity status and waist circumference to define abdominal obesity. Third, many factors related to serum lipid levels among children and adolescents, including physical activity levels, dietary habits, and pubertal status, were not considered in this study. Secular changes in these factors may influence secular trends in serum lipid levels. Further studies should be conducted to examine possible explanations for the observed trends in serum lipids in Beijing, China. Another limitation of the present study is that the trends for specific lipids were not statistically significant among children aged 6–9 years, which might be due to the low statistical power in this group. However, the difference between the two surveys for the total population almost reached statistical significance. On the whole, these comparisons were still meaningful.

In conclusion, our results suggest that adverse trends in serum lipid concentrations were observed among children aged 6–9 years, with the exception of specific lipids, among adolescents aged 10–18 years from several schools in Beijing, China, over the 10-year period between 2004 and 2014. In addition, serum lipid concentration was associated with obesity, and the increased prevalence of obesity is a likely contributor to this adverse serum lipid trend. Thus, adverse trends in obesity and serum lipids levels, and their potential impact on the burdens of long-term CVD, are of great concern. These findings highlight the need to take continuous measures to combat these ominous trends in serum lipid concentrations and obesity.

## ONLINE ONLY MATERIALS

eTable 1. Mean serum lipid concentrations over time among children and adolescents in Beijing from 2004 to 2014 by weight status.

eTable 2. Linear regression analysis of serum lipid concentrations among children and adolescents in Beijing, 2004–2014.

eTable 3. The power in subgroups for which the trends of the serum lipid concentrations were not significant.
