# Supplementary material for: 10-Year Trends in Serum Lipid Levels and Dyslipidemia Among Children and Adolescents From Several Schools in Beijing, China
Source: J Epidemiol. 2016 Dec 5;26(12):637–45. doi: 10.2188/jea.JE20140252 (PMC5121432; doi:10.2188/jea.JE20140252)
Supplement: eTable 1. [file je-26-637-s001.pdf]

eTable 1. Mean serum lipid concentrations over time among children and adolescents in Beijing from 2004 to 2014 by weight status

	TC			TG[a]			HDL-C			Non-HDL-C			LDL-C		
	**2004**	**2014**	***P* value**	**2004**	**2014**	***P* value**	**2004**	**2014**	***P* value**	**2004**	**2014**	***P* value**	**2004**	**2014**	***P* value**
BMI															
Non-overweight	4.04 (0.02)	4.25 (0.02)	<0.001	0.80 (0.01)	0.82 (0.01)	0.044	1.57 (0.01)	1.53 (0.01)	<0.001	2.47 (0.02)	2.72 (0.02)	<0.001	2.20 (0.02)	2.30 (0.02)	<0.001
Overweight	3.94 (0.06)	4.21 (0.05)	<0.001	0.84 (0.04)	0.93 (0.04)	0.016	1.40 (0.02)	1.38 (0.02)	0.577	2.53 (0.05)	2.82 (0.04)	<0.001	2.37 (0.05)	2.40 (0.04)	0.498
Obesity	4.03 (0.06)	4.33 (0.05)	<0.001	1.01 (0.05)	1.16 (0.04)	0.004	1.29 (0.02)	1.33 (0.02)	0.397	2.74 (0.05)	3.01 (0.05)	<0.001	2.51 (0.05)	2.55 (0.04	0.443
WC															
Non-obesity	4.04 (0.02)	4.25 (0.02)	<0.001	0.80 (0.01)	0.81 (0.01)	0.046	1.57 (0.01)	1.53 (0.01)	<0.001	2.47 (0.02)	2.71 (0.02)	<0.001	2.20 (0.02)	2.29 (0.02)	0.001
Abdominal obesity	4.00 (0.04)	4.26 (0.03)	<0.001	0.97 (0.04)	1.07 (0.03)	0.005	1.33 (0.02)	1.34 (0.01)	0.908	2.66 (0.04)	2.92 (0.03)	<0.001	2.44 (0.04)	2.48 (0.03)	0.322

BMI, body mass index; WC, waist circumference; LDL-C, high-density lipoprotein cholesterol; TC, total cholesterol; LDL-C, low-density lipoprotein cholesterol; TG, triglycerides. Non-HDL-C levels equal serum TC levels minus HDL-C. Data are presented as mean (SE). Linear trends in mean serum lipid concentrations were tested using a multivariate linear regression model adjusted for sex and age.

[a] The distribution of TG is skewed. Data are presented as geometric mean (SE)
