# Supplementary material for: 10-Year Trends in Serum Lipid Levels and Dyslipidemia Among Children and Adolescents From Several Schools in Beijing, China
Source: J Epidemiol. 2016 Dec 5;26(12):637–45. doi: 10.2188/jea.JE20140252 (PMC5121432; doi:10.2188/jea.JE20140252)
Supplement: eTable 2. [file je-26-637-s002.pdf]

eTable 2. Linear regression analysis of serum lipid concentrations among children and adolescents in Beijing, 2004-2014

	TC	TG[#]	HDL-C	Non-HDL-C	LDL-C
2004	0 (Reference)	0 (Reference)	0 (Reference)	0 (Reference)	0 (Reference)
2014[b]	0.197 (0.150 to 0.244)	0.035 (0.023 to 0.047)	-0.075 (-0.097 to -0.053)	0.272 (0.229 to 0.315)	0.108 (0.067 to 0.148)
2014[c]	0.195 (0.147 to0.244)	0.022 (0.010 to 0.033)	-0.048 (-0.070 to -0.027)	0.243 (0.200 to 0.287)	0.077 (0.036 to 0.118)
2014[d]	0.195 (0.146 to 0.244)	0.019 (0.007 to 0.031)	-0.043 (-0.065 to -0.022)	0.238 (0.194 to 0.283)	0.071 (0.030 to 0.113)

HDL-C, high-density lipoprotein cholesterol; TC, total cholesterol; LDL-C, low-density lipoprotein cholesterol; TG, triglycerides. Non-HDL-C levels equal serum TC levels minus HDL-C. Data are presented as β coefficients (95% CI).

[a] The distribution of TG is skewed. Data are presented as geometric means (SE).

[b] Adjusted for sex and age.

[c] Adjusted for sex, age, and weight status defined by BMI.

[d] Adjusted for sex, age, and abdominal obesity.
