# Supplementary material for: 10-Year Trends in Serum Lipid Levels and Dyslipidemia Among Children and Adolescents From Several Schools in Beijing, China
Source: J Epidemiol. 2016 Dec 5;26(12):637–45. doi: 10.2188/jea.JE20140252 (PMC5121432; doi:10.2188/jea.JE20140252)
Supplement: eTable 3. [file je-26-637-s003.pdf]

eTable 3. The power in subgroups for which the trends of serum lipid concentrations were not significant

	6-9 years	
	**Boys**	**girls**
TG	0.85	0.85
HDL-C	0.50	0.50
LDL-C	0.50	0.50
High TG	0.20	0.27
High non-HDL-C	0.98	1.00
High LDL-C	0.15	0.50

HDL-C, high-density lipoprotein cholesterol; TC, total cholesterol; LDL-C, low-density lipoprotein cholesterol; TG, triglycerides. Non-HDL-C levels equal serum TC levels minus HDL-C.
